# Altered hypoxia inducible factor regulation in hereditary haemorrhagic telangiectasia

**DOI:** 10.1038/s41598-022-09759-9

**Published:** 2022-04-07

**Authors:** Anna Wrobeln, Tristan Leu, Jadwiga Jablonska, Urban Geisthoff, Stephan Lang, Joachim Fandrey, Freya Droege

**Affiliations:** 1 Institute of Physiology, University Hospital Essen, University of Duisburg-Essen, Hufelandstraße 55, 45122 Essen, Germany; 2 Translational Oncology, Department of Otorhinolaryngology, University Hospital Essen, University Duisburg-Essen, Hufelandstaße 55, 45147 Essen, Germany; 3 Department of Otorhinolaryngology, Head and Neck Surgery, University Hospital Essen, University of Duisburg-Essen, Hufelandstraße 55, 45122 Essen, Germany; 4 Department of Otorhinolaryngology, Head and Neck Surgery, University Hospital Marburg, Philipps-Universität Marburg, Baldingerstrasse, 35043 Marburg, Germany

**Keywords:** Diseases, Medical research, Molecular medicine

## Abstract

Patients with hereditary haemorrhagic telangiectasia (HHT), also known as Rendu–Osler–Weber syndrome, suffer from the consequences of abnormal vessel structures. These structures can lead to haemorrhages or shunt effects in liver, lungs and brain. This inherited and rare disease is characterized by mutations affecting the transforming growth factor-β (TGF-β)/Bone Morphogenetic Protein (BMP) pathway that results in arteriovenous malformations and studies indicate an impaired immune response. The mechanism underlying this altered immune response in HHT patients is still unknown. TGF-β interacts with hypoxia inducible factors (HIF), which both orchestrate inflammatory and angiogenic processes. Therefore, we analysed the expression of HIF and related genes in whole blood samples from HHT patients. We could show significantly decreased expression of HIF-1α on the mRNA and protein level. However, commonly known upstream regulators of HIF-1α in inflammatory responses were not affected, whereas HIF-1α target genes were significantly downregulated. There was no correlation between *HIF1A* or *HIF2A* gene expression and the severity of HHT detected. Our results represent a rare case of HIF-1α downregulation in a human disease, which underlines the relevance of HIFs in HHT. The study indicates an interaction of the known mutation in HHT and the dysregulation of HIF-1α in HHT patients, which might contribute to the clinical phenotype.

## Introduction

Patients with hereditary haemorrhagic telangiectasia (HHT), also known as Rendu–Osler–Weber syndrome, suffer from recurrent hemorrhages as the consequences of abnormal vessel structures. This inherited, rare disease is characterized by multisystemic arteriovenous malformations, particularly in liver, lung, intestine and brain^1^. The underlying mutations affect genes from the transforming growth factor-β (TGF-β)/Bone Morphogenetic Protein (BMP) pathway, thus impairing its signalling^2^. TGF-β is a regulatory cytokine involved in cell growth, apoptosis, smooth muscle cell differentiation, vascular remodeling and the immune response^3^. In addition to vascular malformations, few studies indicate impaired immune response in HHT patients, the precise mechanism of the immune dysregulation is still unknown^4,5^. In this context, several publications indicate an interaction of TGF-β with hypoxia inducible factors (HIFs)^6–9^. Further, Endoglin (the gene responsible for HHT type 1) expression is regulated by transcriptional cooperation between the HIF and TGF-β pathways^10^. In general, HIFs are responsible for cellular oxygen sensing and the further adaption to hypoxic conditions via the induction of target genes involved in vascularization, angiogenesis, cell metabolism, cell survival and tumourgenesis^11^. HIFs are heterodimeric transcription factors, which consist of an oxygen sensitive HIF-α subunit, with three identified isoforms [HIF-1α, HIF-2α and HIF-3α (with a minor role)], and a HIF-β subunit^12^. Both subunits are expressed constitutively but the expression pattern of HIF-1α and HIF-2α differ: HIF-1α is ubiquitously expressed among all tissues, while HIF-2α expression is limited for example to endothelium, kidney, pancreas, liver, heart, lungs, intestine, brain and immune cells^13–15^. Regulation of HIF-α expression by cellular oxygen sensing occurs mainly by a post-translational mechanism. Under normoxic conditions, HIF-α subunits are hydroxylated by oxygen-sensitive hydroxylase enzymes [prolyl hydroxylase domain-containing enzymes (PHDs)], afterwards ubiquitinylated through the von Hippel–Lindau protein ubiquitin ligase complex, and degraded in the proteasome^16^. Since PHDs require oxygen for their enzymatic activity, hypoxic conditions result in reduced hydroxylase activity of their substrates, thus leading to HIF-α subunits accumulation, increased translocation to the nucleus, and augmented formation of a heterodimeric complex with the HIF-β subunit.These dimers bind to genes with hypoxia response elements and thus promote cell responsiveness to diminished oxygen availability^17^. As a type of self-regulatory system, PHDs are HIF target genes and therefore upregulated following HIF activation. HIFs accumulate not only in hypoxia but also in inflammation and perform key functions in the immune response^18,19^. Due to the paramount role played by HIFs in modulation of both angiogenesis and the immune response, as well as in the transcriptional regulation of Endoglin, VEGF and Erythropoietin, gaining insight into HIFs expression profile in HHT may help characterization of the pathogenic mechanisms in this rare disease^20^. For the first time, this study has analysed HIFs and their corresponding target genes in whole blood samples from HHT patients.

## Results

### Characteristics of patients with HHT and non-HHT control individuals

The study included 66 patients with 29 (44%) males and 37 (56%) females. The age range was 38–66 years (m ± SD: 52 ± 14 years). Genetic testing was performed in 23 (35%) patients with most patients suffering from HHT type 1 (n = 7.11%). More than 90% of patients suffered from epistaxis and multiple typical telangiectasia, and reported a positive family history for HHT (Table 1). Patients with HHT and non-HHT donors were of comparable age and sex.

**Table 1:** Clinical characteristics of 66 patients with hereditary haemorrhagic telangiectasia.

			All patients n = 66 (100)	Men n = 29 (44)	Women n = 37 (56)
Age		Years	52 ± 14 (min: 18, max: 82)	56 ± 14	48 ± 14
		Missing	0	0	0
Genetic testing		Yes	23 (35)	7 (24)	16 (43)
		No	43 (65)	22 (76)	21 (57)
		Missing	0	0	0
Mutated gene		HHT 1	7 (11)	0	7 (19)
		HHT 2	4 (6)	2 (7)	2 (5)
		Others	2 (3)	1 (3)	1 (3)
		No mutation detected	1 (2)	0	1 (3)
		Missing	9 (39)	4 (57)	5 (31)
Positive FH		Yes	60 (91)	26 (90)	34 (92)
		No	5 (8)	3 (10)	2 (5)
		Not known	1 (2)	0	1(3)
		Yes	64 (97)	29 (100)	35 (95)
TAE		No	2 (3)	0	2 (5)
		Missing	0	0	0
Visceral lesions	GI	Yes	22 (33)	12 (41)	10 (27)
		No	27 (41)	11 (38)	16 (43)
		Not known	17 (26)	6 (21)	11 (30)
	PAVM	Yes	33 (55)	12 (41)	21 (57)
		No	29 (44)	15 (52)	14 (38)
		Not known	4 (6)	2 (7)	2 (5)
	HVM	Yes	17 (26)	6 (21)	11 (30)
		No	34 (52)	16 (55)	18 (49)
		Not known	15 (23)	7 (24)	8 (22)
	CVM	Yes	8 (12)	5 (17)	3 (8)
		No	40 (61)	16 (55)	24 (65)
		Not known	18 (27)	8 (28)	10 (27)
Epistaxis		Yes	64 (97)	28 (97)	36 (97)
		No	2 (3)	1 (3)	1 (3)
		Missing	0	0	0
ESS		Score	5.5 ± 2.3 (min: 0, max: 9.26)	5.4 ± 2.3	5.5 ± 2.4
		Missing	0	0	0
Haemoglobin		g/dl	12.5 ± 2.7	13.4 ± 2.7	11.8 ± 2.5
		Missing	0	0	0

*TAE* teleangiectasia, *FH* family history, *GI* gastrointestinal bleedings, *PAVM* pulmonary arteriovenous malformations, *HVM* hepatic vascular malformations, *CVM* cerebral vascular malformations, *ESS* Epistaxis Severity Score with a scale from 1 = mild epistaxis to 10 = severe epistaxis. Data is shown in number of patients [(n) and (percentage)] or mean ± SD.

### Altered gene transcription of HIF and hypoxia related genes in whole blood of HHT patients

**Figure 1:**
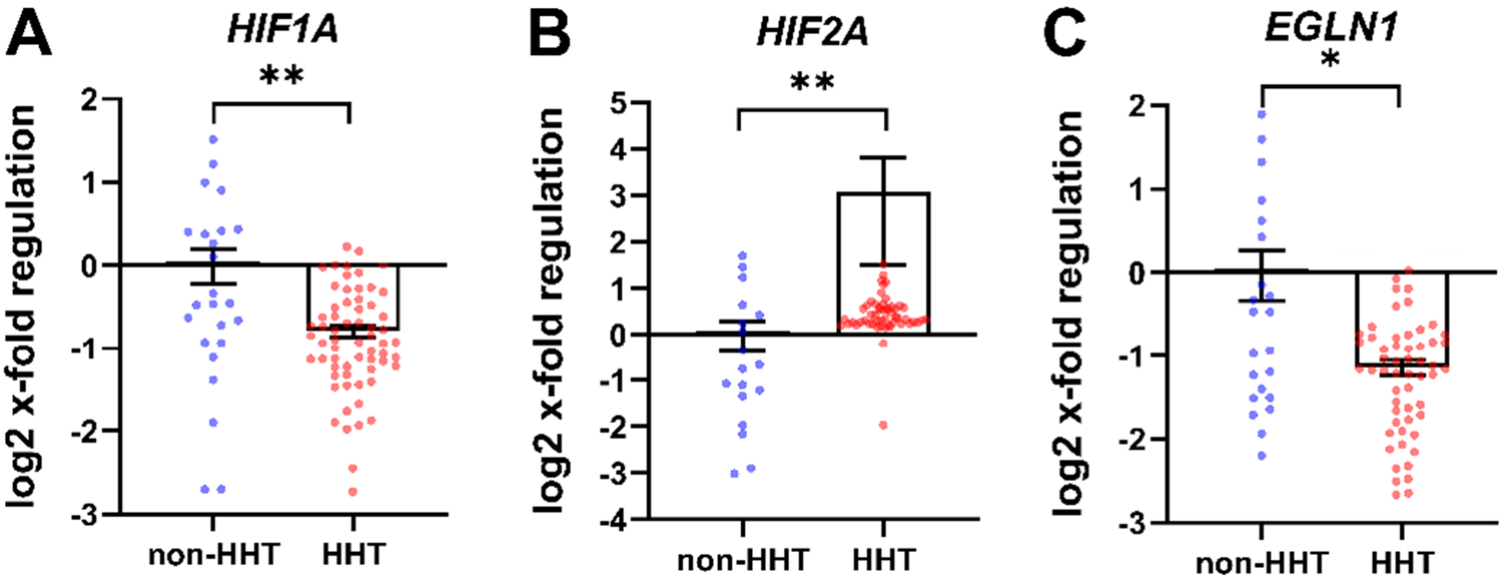
Hypoxia related gene transcription of whole blood in HHT compared to non-HHT controls. Patients with HHT showed decreased *HIF1A* (**A**) and increased *HIF2A* (**B**) gene expression. The HIF-1α target gene *EGLN1* (**C**) is significantly decreased in HHT patients. Data are presented as log2 x-fold regulation in mean ± SEM. The Mann Whitney U test was used to compare the groups, *p < 0.05, **p < 0.01. n (non–HHT) = 26, n (HHT) = 66).

According to RT-PCR analysis, *HIF1A* gene expression was reduced by approximately 50% in blood cells of HHT patients compared to non-HHT controls (Fig. 1A). In contrast, *HIF2A* gene expression was upregulated in HHT patients compared to non-HHT controls (Fig. 1B). The HIF-1α target gene *EGLN1* (encoding PHD2) (Fig. 1C), which is part of the hypoxic response, was also reduced by 50% in HHT patients compared to non-HHT controls.

### Decreased gene transcription of inflammation related genes in whole blood of HHT patients

**Figure 2:**
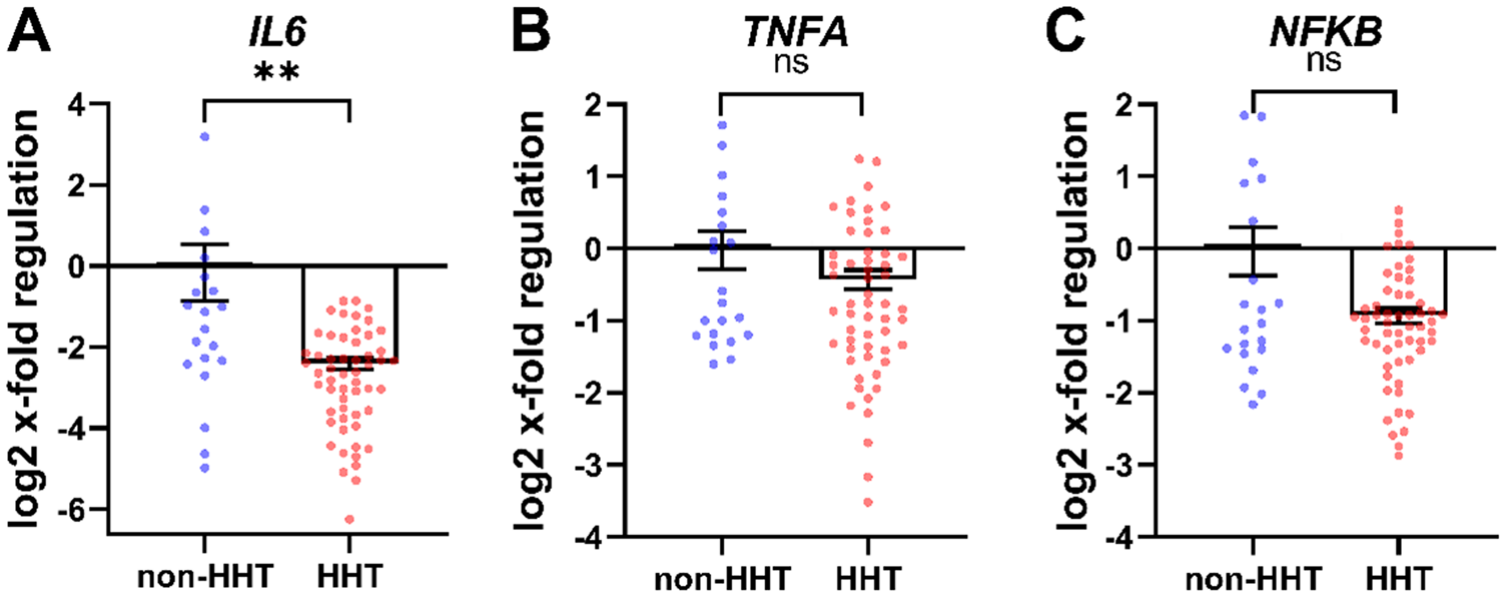
Inflammation related gene transcription of whole blood in HHT compared to non-HHT controls. HIF-1α target gene *IL6* (**A**) was significantly downregulated in HHT patients, whereas *TNFA* (**B**) and *NFKB* (**C**), the upstream regulator of HIF-1α, showed no difference in gene expression. Data are presented as log2 x-fold regulation in mean ± SEM. Mann Whitney test was used to compare the groups, ***p < 0.001, ns = not significant. n (non–HHT) = 26, n (HHT) = 66).

To confirm the biological relevance of altered HIF-regulation, the expression of different inflammation-related genes was analysed. The expression of HIF-1α target gene *IL6* was significantly decreased in HHT patients compared to non-HHT controls (Fig. 2A). *TNFA* gene expression (Fig. 2B) and the gene expression of *NFKB* showed no significant difference between the analysed groups (Fig. 2C).

### Gene transcription of angiogenesis related genes in whole blood of HHT patients is not affected

**Figure 3:**
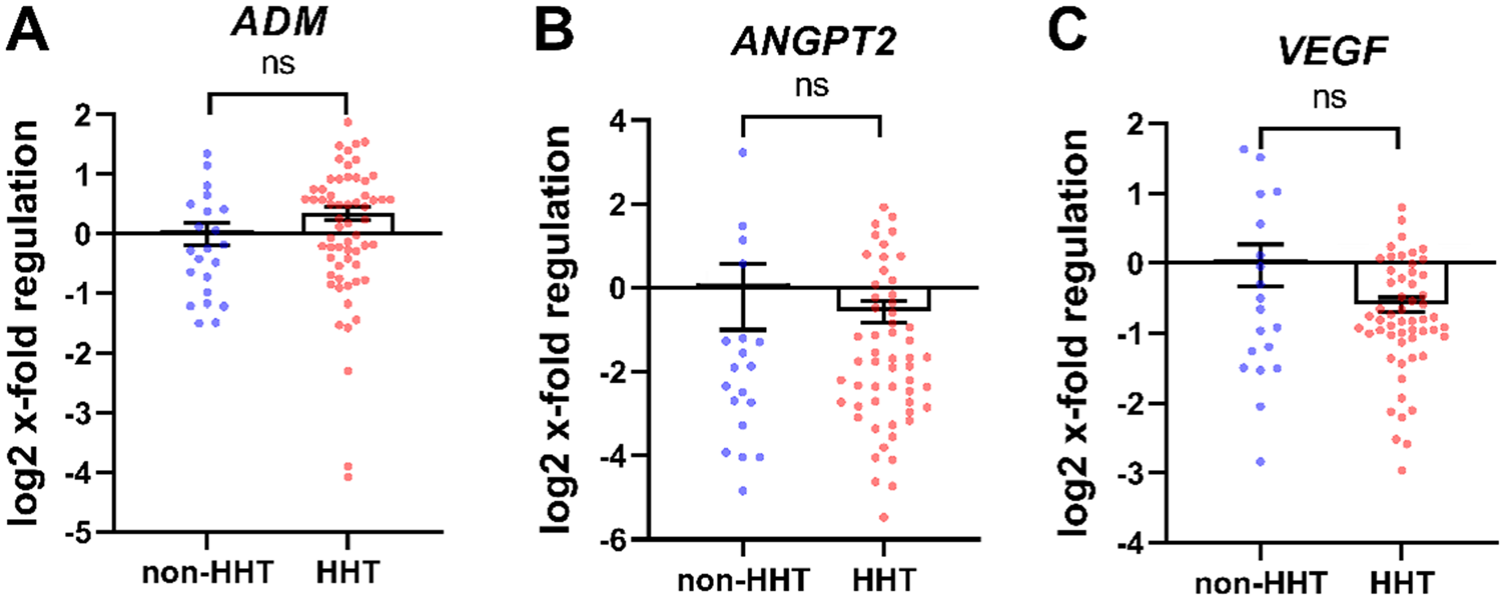
Angiogenesis related gene transcription of whole blood in HHT compared to non-HHT controls. Patients with HHT showed no differences in the regulation of HIF target genes *ADM* (**A**). *ANGPT2* (**B**) and *VEGF* (**C**). Data are presented as log2 x-fold regulation in mean ± SEM. The Mann–Whitney U test was used to compare the groups, ns = not significant. n (non-HHT) = 26, n (HHT) = 66.

Further analysis was performed of HIF-regulated genes with biological relevance to angiogenesis. The expression of HIF-1 target gene *ADM* (Fig. 3A), *ANGPT2* (Fig. 3B) and *VEGF* (Fig. 3C) was not affected, comparing HHT patients and non-HHT control individuals.

### Decreased HIF-1α protein in whole blood of HHT patients

**Figure 4:**
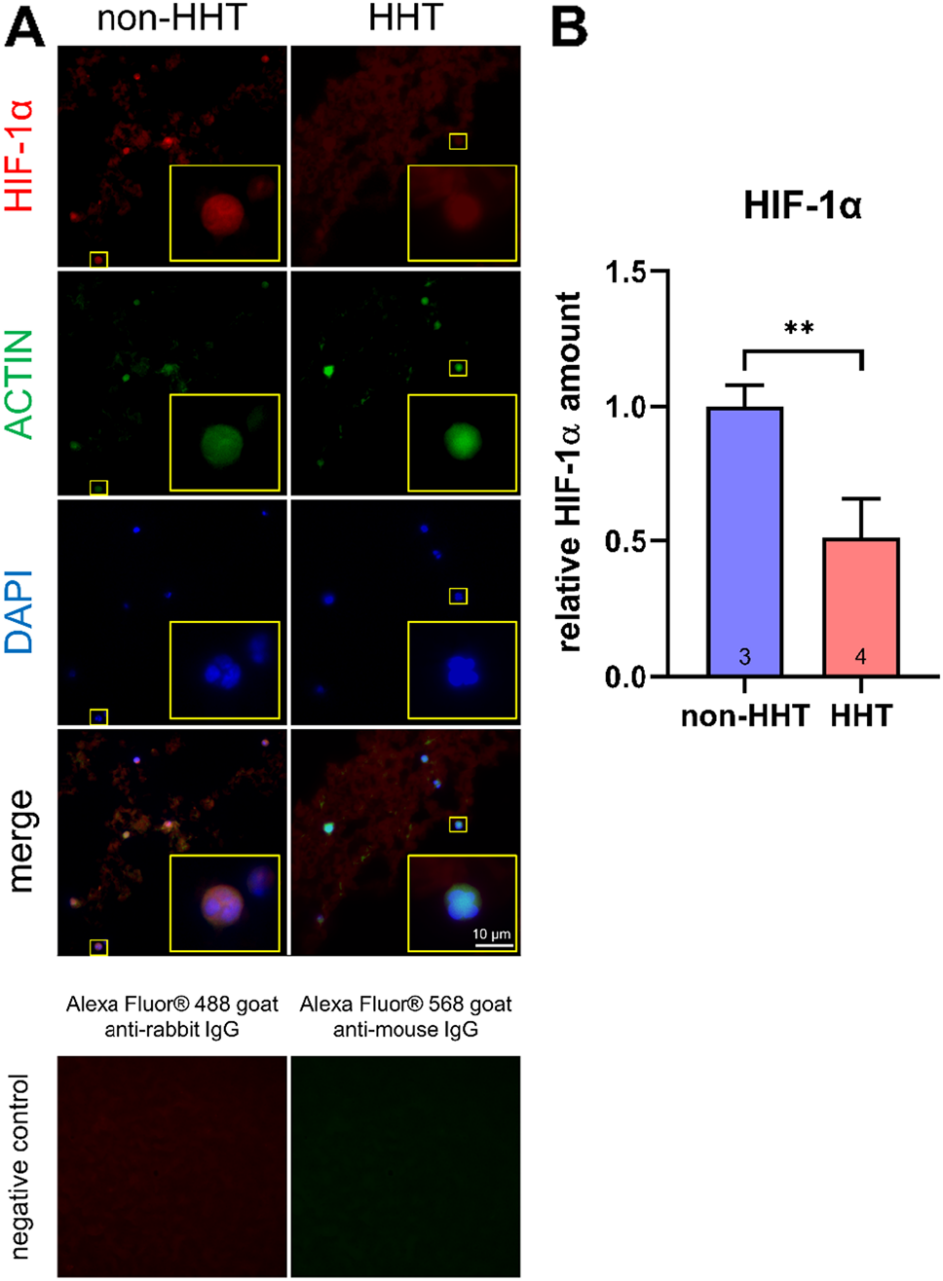
HIF-1α protein immunostaining in whole blood. Whole blood of HHT patients and non-HHT controls were immunostained. Examples of pictures from cells are shown in (**A**). HIF-1α is shown in red, ACTIN in green and the nuclei in blue. As negative control, staining with secondary antibody only is shown for non-HHT controls. Quantification of HIF-1α amount is shown in (**B**). HHT patients showed significantly less amounts of HIF-1α. Unpaired t-test was applied to determine significantly differences between the groups. Data are shown as mean ± SD, **p < 0.01.

To gain insight on the effects of downregulated HIF1A gene expression at the protein level, we assessed HIF-1α protein levels in blood cells of HHT patients and non-HHT control individuals by immunostaining. HIF-1α protein level was significantly reduced by about 50% in blood cells of HHT patients compared to non-HHT controls (Fig. 4).

### No correlation between HHT patients, HIF gene expression and the Epistaxis Severity Score

**Figure 5:**
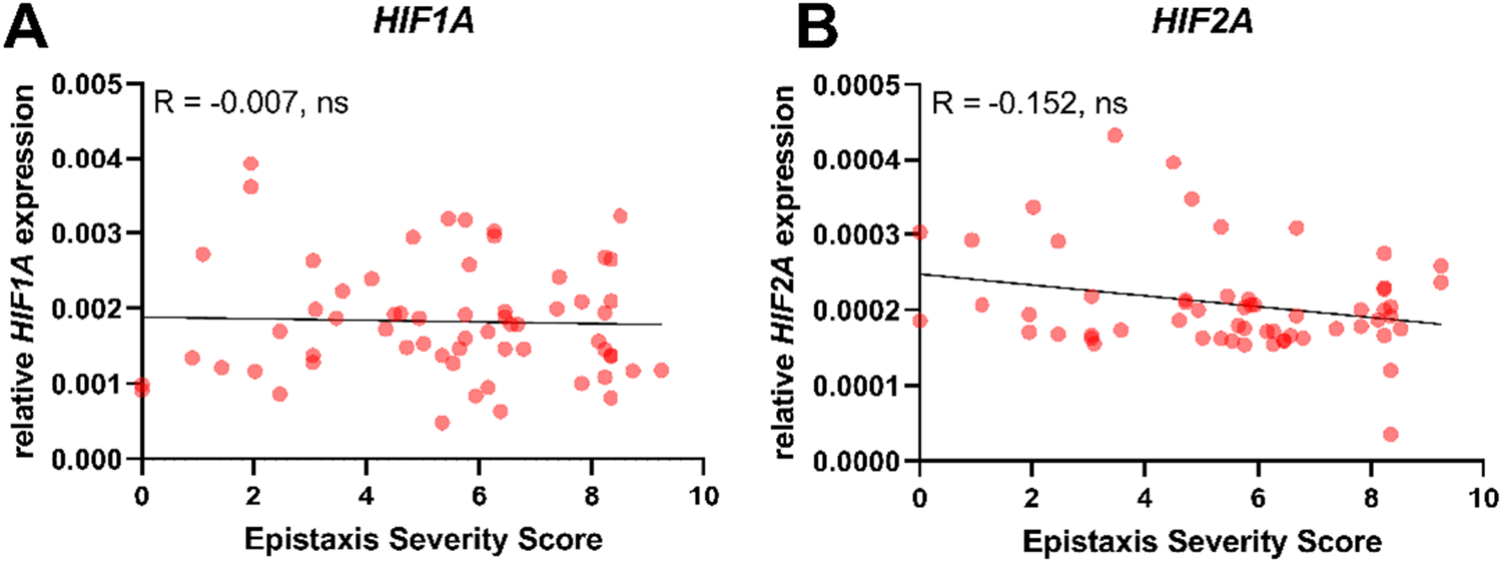
Correlation of HIF and Epistaxis Severity Score. Individual HHT patients’ Epistaxis Severity Score was correlated to individual relative *HIF1A* gene transcription (n = 62) (**A**) or individual relative *HIF2A* gene expression (n = 58) (**B**). Gene expression was calculated as 2-ΔCT expression with respect to *ACTB.* No significant correlation was observed. Data are presented as individual values; Spearman R test was used to assess the correlations. *ns* not significant.

There was no significant correlation between *HIF1A* gene expression and the individual Epistaxis Severity Score of HHT patients (Fig. 5A). Furthermore, the correlation of HHT patients’ individual Epistaxis Severity Score and *HIF2A* gene expression showed no significant correlation (Fig. 5B).

## Discussion

HHT patients suffer from vascular disorders, which lead to recurrent bleeding, as well as immunological abnormalities are indicated in few studies^1,4,5,21^. Angiogenesis and inflammation are closely related processes and HIF is a key player in both of them. HIF activation in both hypoxia and inflammation regulates the transcription of several genes related to angiogenesis, metabolism, cell proliferation and apoptosis^17^. For the first time, this study provides evidence that HHT might alter expression of HIF and, potentially, of some HIF-regulated downstream genes.

Recurrent bleeding cause lower haemoglobin levels (11.8 ± 2.5 g/dl, Table 1), which reduces the oxygen supply to the tissues resulting in hypoxia. Moreover, this hypoxia should be latent but global throughout the body. This phenomenon led us to assume that the amount of HIF-1α protein would have increased or—with respect to the mRNA level—at least not altered^22^. Unexpectedly, RNA expression analysis in whole blood revealed significantly decreased HIF-1α expression in HHT patients’ leukocytes (Fig. 1A). Even target genes related to cellular hypoxic response of HIF-1α showed decreased or unchanged expressions in HHT patients (Fig. 1C) which is consistent with a reduction of HIF-1α also at the protein level. We succeeded in performing HIF-1α staining in whole blood cryosections of human subjects and found reduced amounts of HIF-1α protein in HHT-patients’ leucocytes (Fig. 4).

Obviously, analysis of whole blood RNA and protein expression of HIF-α in particular will provide data for leucocyte expression because they are the only nucleated cells in the blood. Gene expression in leucocytes from HHT patients is also of interest due to their impaired immune response. Thus, it was of interest to see inflammation related HIF-1α target gene *IL6* was significantly downregulated (Fig. 2A). On the other hand, there was no change in the expression of *TNFα* as a general marker of acute inflammation in HHT patients (Fig. 2B). This finding was corroborated by unaltered *NFKB* expression in HHT patients (Fig. 2C). This, again, is of interest with respect to HIF-1α expression because NFKB is believed to act upstream of HIF-1α gene expression; based on our result, no evidence exists for a role of NFKB to mediate the reduced expression of HIF-1α at the transcript level in HHT^19,23^.

As mentioned above, many studies describe abnormalities in the immune response of HHT patients, to the extent that patients had milder symptoms during COVID infections or are less prone to tumour development compared to the non-diseased population^24,25^. Both the development of symptoms in COVID-19 and the development of some tumours as an effect of chronic inflammation closely depend on the immune response, which in part is related to the increased expression of HIF target genes^26,27^. Of note, a very recent work by Taniguchi-Ponciano et al*.* showed that critically ill COVID-19 patients had significantly increased HIF-1α expression in leukocytes^28^. Thus, reduced HIF-1α expression in immune cells may protect HHT patients from the exacerbating activation of the immune system, as it occurs in COVID-19 infections.

How might reduced HIF-1α affect leukocyte function in HHT? Impaired migration of human HHT monocytes was found in a study by Han et al.^29^. In 2003, one of the first studies on the role of HIF-1α in immune cells by Cramer et al*.* already reported myeloid cell aggregation, motility and invasiveness to be impaired in mice with a conditional *Hif1α* knockout. Similarly, Kojima et al*.* suggested that HIF-1α deficiency depressed the function of cytotoxic T-lymphocytes and blocked B-cell development in bone marrow^30,31^. The observation by Han et al.^29^ may therefore now be understood as an effect of reduced HIF-1α. This may have contributed to an impaired motility. This is in agreement with our previous studies, where we could demonstrate impaired motility of mouse macrophages with a conditional *Hif1α* knock out^32^.

Of note, *HIF2A* expression showed the opposite effect from what we observed with *HIF1A*, namely an increased gene expression in HHT patients (Fig. 1B). In mice, the conditional knockout of *Hif2a* in cells of the myeloid lineage resulted in increased recruitment of neutrophils to deeper layers of the colon during colitis^33^. One could speculate that HHT immune cells with increased HIF-2α might react in the opposite way and not be able to migrate into tissues and activate the common immune response. Obviously, we have to take into account that HIF-1α is expressed in all immune cell types, while HIF-2α is more selectively found in neutrophils and NK cells under hypoxic conditions as reviewed in Palazon et al*.*^19^. Thus, differential composition of immune cells and in particular the higher numbers of neutrophils and NK-cells in HHT patients may explain the increase in the overall higher leukocyte *HIF2A* expression^21^.

Following several studies’ descriptions of the influence of immune cells on angiogenesis and the vascular abnormalities in HHT patients, we analysed HIF target genes influencing the angiogenesis^34–37^. Surprisingly, changes in angiogenesis associated genes were not detected in HHT patients’ leukocytes (Fig. 3). It appears that, at the very least, blood leucocytes do not contribute to the vascular phenotype in HHT by expressing and secreting significantly higher levels of angiogenic molecules. This stands in contrast to the report of increased plasma VEGF levels in HHT patients by Cirulli et al*.* and Sadick et al*.* but may simply indicate that tissues other than those that are immune secrete VEGF in this disease^38,39^.

As mentioned above, key molecules in HHT are components of the TGF-β/BMP-pathway^2^. Caused by the haploid deficiency of the TGF-β/BMP pathway, TGF-β levels are elevated in the plasma of HHT patients^39^. Following previous studies, this should lead to an increase in HIF-1α regulation^6–8^; thus TGF-β itself is unlikely to be the reason for the reduction of HIF-1α in HHT patients. However, when the impaired TGF-β/BMP pathway occurring in HHT was stimulated by suppressing TGF-β signalling with TGF-β antagonists, rat kidney cells showed decreased HIF-1α expression^40^. We therefore assume that the full functionality of the TGF-β/BMP pathway is required for both HIF-1α expression and protein accumulation. To prove this hypothesis, further studies should aim to reconstitute TGF-β signalling in HHT leucocytes in order to increase *HIF1A* expression to normal levels.

To evaluate the severity of the clinical presentation of the disease, clinicians are still searching for suitable biomarkers for HHT, which are missing in daily clinical use^41–43^. To that end, we correlated the results of *HIF1A* and *HIF2A* gene expression with the clinically determined Epistasis Severity Score of each individual HHT patient. Unfortunately, a significant correlation between disease severity and *HIF* gene expression was not detected (Fig. 5) which may indicate that the situation is more complex and cannot be fully explained by such a correlation. It was demonstrated that TGF-β/BMP pathway members and HIF-1α interact and form a multi-protein complex on the promoter of the TGF-β co-receptor (endoglin) and through that mechanism, influence vascular remodelling and angiogenesis^9^. This multi-protein complex might be disturbed in HHTs because of reduced HIF-1α. In addition to the known mutations in endoglin, HIF-1α could contribute and aggravate defects in TGF-β signalling leading to dysregulated angiogenesis in HHT patients.

Taken together, our findings show that the regulation of HIF-1α in HHT patients does not support the canonical regulation during hypoxia or inflammation. The results point towards a downregulation caused by the haploid impairment of the TGF-β/BMP pathway, but the role of different other factors remain to be elucidated While reduced expression of the *HIF1A*-gene and HIF-1α protein has, to our knowledge, never been described in any disease, future studies might reveal the underlying mechanism.

## Materials and methods

### Selection of participants and clinical parameters

The study was approved by the ethics committee of the University Duisburg-Essen (20-9162-BO). Participants were informed and gave written consent in accordance with the Declaration of Helsinki. The study is registered at Clinical trials.gov (ID NCT04469517).

Adult patients with confirmed HHT (fulfilled at least three out of four Curaçao Criteria and/or had a positive genetic testing) were included in this prospective study^1,44^. Alongside patients’ characteristics such as age and gender, factors that might also affect HIF production were documented. HIF affecting parameters include smoking habits, days spent at height (> 1000 m above sea level) and the median weekly time spent doing sports. The documented HHT related clinical parameters include frequency, duration and intensity of epistaxis, need for medical attention, transfusions and signs of anaemia (haemoglobin level), family history, mucocutaneous telangiectases and visceral arterio-venous malformations especially involving the liver, lungs, gastrointestinal tract and central nervous system. The recurrent nosebleeds were described using the Epistaxis Severity Score (ESS)^45^. In all cases, whole blood samples were taken from patients peripheral arm veins. All experiments were compared with blood from a non-HHT control group. The non-HHT control group exists of 26 volunteers (15 men, 11 women) recruited from university employees (mean age: 40 ± 13; min: 21, max: 63). The number of patients and non-HHT donors is listed in the figures for each experiment.

### RNA preparation and RT-PCR of whole blood

RNA from whole blood was isolated using the PAXgene^®^ blood RNA collection system (Qiagen, Mississauga, Canada) according to the manufacturer’s protocol. 2.5 ml of whole blood was drawn from HHT patients or non-HHT controls into PAXgene^®^ blood RNA tubes (Qiagen, Mississauga, Canada) and stored at – 20 °C.

Complementary DNA (cDNA) was synthesised using one µg of RNA and Moloney murine leukemia virus reverse transcriptase (Promega, Walldorf, Germany) according to the manufacturer’s instructions. Quantification of gene expression was performed by real-time PCR (RT-PCR) with SYBR green fluorescent dye (Eurogentec, Verviers, Belgium) and the CFX96™ Real Time System (Bio-Rad Laboratories GmbH, Munich, Germany). cDNA was amplified by 40 cycles of 95 °C for 15 s and 60 °C for 90 s with gene specific primers (Table 2) and normalized to *ACTB* (actin). Primer specificity was checked by Primer-BLAST and confirmed by size analysis of the PCR amplicons^46^. Expression was calculated with the 2-ΔCT method for statistical analysis and set as an induction relative to non-HHT controls in figures.

**Table 2:** Human primer sequences.

Gene	5′Primer	3′Primer
*ACTB*	TCACCCACACTGTGCCCATCTACGA	CAGCGGAACCGCTCATTGCCAATGG
*ADM*	AGTCGTGGGAAGAGGGAACT	ATCCGGACTGCTGTCTTCGG
*ANPT2*	AACTTTCGGAAGAGCATGGAC	CGAGTCATCGTATTCGAGCGG
*HIF-1α*	TCACTGGGACTATTAGGCTCAGGT	CTCCATTACCCACCGCTGAA
*HIF-2α*	CGGAGGTGTTCTATGAGCTGG	ASCTTGTGTGTTCGCAGGAA
*IL-6*	TGCATCTAGATTCTTTGCCTTTTT	CCACTCACCTCTTCAGAACGAA
*NFκB*	AACAGAGAGGATTTCGTTTCCG	TTTGACCTGAGGGTAAGACTTCT
*EGLN1*	CCAGCTTCCCGTTACAGT	GCACGACACCGGGAAGTT
*TNFα*	GGCGTGGAGCTGAGAGATAAC	GGTGTGGGTGAGGAGCACAT
*VEGF*	GCAAGACAAGAAAATCCCTGTGGGCC	CCGCCTCGGCTTGTCACA

Primer sequences of specific PCR products used for RNA quantifications of human whole blood via RT-PCR.

### HIF-1α staining of whole blood

Following withdrawal, blood was immediately transferred in to PHEM buffer (3 mmol PIPES, 1 mmol/l HEPES, 0.5 mmol/l EGTA, 0.25 mmol/l MgCl_2_, 3.5 mmol/l KCl, pH = 7.2) containing 4% paraformaldehyde and 2.5% glutaraldehyde. After 1 h of fixation, cells were centrifuged (RT, 10 min, 1500 rcf) and the pellet was cryoconserved in Tissue-Tek^®^ O.C.T.™ Compound (Sakura Finetek, Netherlands). 10 µm sections were stained with primary antibodies against HIF-1α (BD Transduction Laboratories™, Switzerland) and ACTIN (Sigma-Aldrich, Inc.). For secondary antibodies Alexa Fluor^®^ 488 goat anti-rabbit IgG (Life Technologies) and Alexa Fluor^®^ 568 goat anti-mouse IgG (Invitrogen AG) were used. To visualize nuclei, cells were covered with 2-[4-(Aminoiminomethyl)phenyl]-1H-Indole-6-carboximidamide hydrochloride (DAPI)-added Mowiol. At least 20 cells per subject were quantified.

### Statistics

GraphPad Prism 8.4.3. (GraphPad Software Inc., La Jolla, CA, USA) was used for statistical analysis. After elimination of outliers (ROUT-test), the groups were analysed for Gaussian distribution using the D’Agostino–Pearson test. An unpaired t-test was applied to Gaussian distributed data and Mann–Whitney U test to non-Gaussian distributed data. Statistical significance is displayed as *(p < 0.05), **(p < 0.01), ***(p < 0.001) or ****(p < 0.0001).

### Informed consent statement

Informed consent was obtained from all subjects involved in the study.

## Limitations of the study

As in many studies of rare diseases, we have to mention the limited size of the investigated patient cohort. Regarding gene expression only a small selection of HIF target genes was analysed, more genes could complement this.

## Data Availability

The datasets generated during and/or analysed during the current study are available from the corresponding author.
